# Social venue range and referral chain impact: Implications for the sampling of hidden communities

**DOI:** 10.1371/journal.pone.0181494

**Published:** 2017-08-23

**Authors:** Benjamin Cornwell, John A. Schneider

**Affiliations:** 1 Department of Sociology, Cornell University, Ithaca, New York, United States of America; 2 Departments of Medicine & Public Health Sciences, University of Chicago, Chicago, Illinois, United States of America; University of Cyprus, CYPRUS

## Abstract

**Background:**

It has been argued that the success of respondent-driven sampling (RDS) in generating unbiased estimates for epidemiologic outcomes depends on participants’ abilities to generate long referral chains. While this is thought to depend on the number of people participants know in the target population, this idea is rarely tested. Furthermore, little attention has been paid to the role of other aspects of social connectedness in recruitment, such as participants’ involvement in local clubs and venues.

**Methods:**

We examine whether the recruitment potential of young Black men who have sex with men (YBMSM) depends on (1) their social network size and (2) their affiliations with a variety of sex venues across geographic areas. We analyze data from a 2014 RDS study of 598 YBMSM on the south side of Chicago.

**Results:**

Both a participant’s personal network size and the number of different types of sex venues with which he was affiliated were significantly positively associated with (1) the total number of recruits in the participant’s prospective referral chains and (2) the lengths of those chains. However, only venue affiliation remain significantly associated with recruitment potential in the multivariate model.

**Conclusions:**

The success of RDS in generating valid samples may depend more on recruiting participants who are involved in multiple venues within the community (i.e., their affiliation networks) than on recruiting those who have large personal social networks.

## Background

Due to the concealed nature of “risky” health-related behaviors, the use of chain-referral sampling is increasingly common in epidemiology [1, 2]. This involves relying on respondents to recruit new participants. Respondent-driven sampling (RDS) is a more rigorous form of chain-referral sampling[1, 3, 4, 5, 6]. With RDS, the researcher keeps track of referral linkages among participants and adjusts for the influence of well-connected participants on the composition of the sample. RDS is widely utilized in public health studies, mostly in an attempt to generate samples of people for which official rosters or population-based samples are unfeasible, such as men who have sex with men (MSM) [7, 8, 9, 10, 11].

The ability of RDS to generate unbiased estimates depends on the identification of initial “seeds”—and their social contacts—whose recruitment efforts yield long referral chains. Longer chains that involve numerous successive waves of recruitment produce less clustered referral patterns, thus more closely representing the target population[1, 12]. Unfortunately, the RDS process does not always yield long or expansive referral networks, which can result in estimation bias [9, 13, 14, 15]. For example, if seeds recruit only their close friends, and those friends recruit only their close friends, then the final sample will be a highly clustered (or dense) group in which many of the recruits know each other.

The factors that affect how well the chain-referral process performs are not yet well understood. Some studies have examined individual-level factors that are associated with seeds’ recruitment abilities [16, 17]. RDS studies typically assume that the number of people in the target population (e.g., MSM) that a given participant knows (i.e., his or her personal social network size) is a key factor that drives that participant’s recruitment success [1, 16]. This paper examines referral chains in a group of MSM to determine if organizational, as opposed to individual, social network factors also play a role in this recruitment process.

In this study, we explore the idea that exposure to and encounters with non-network members within the local community may also influence recruitment efforts and thus RDS sampling. Our motivation comes from research on the role that sex-related spaces play in linking MSM to each other, often anonymously[18, 19]. This work emphasizes that people are connected to similar others not only through personal networks, but also through large spaces that cater to others who share similar sexual orientations. Culturally linked spaces are sometimes referred to as sex “venues,” or sex “marketplaces” [20, 21, 22, 23]. These spaces include bars, clubs, bookstores, bathhouses, and other places that facilitate connections among MSM patrons [21]. Large geographic areas, like cities and neighborhoods, typically have numerous venues. Individuals’ connections to these venues affects their risk of contracting or spreading STIs such as HIV[18, 24, 25, 26, 27, 28, 29, 30]. People who are connected to multiple such venues and/or different types of venues are exposed to a larger pool of contacts, and those who serve as “bridges” between different types of venues play a greater role in spreading infection between different populations[31]. Here, we focus on the possibility that MSM who are affiliated with multiple types of sex venues have access to larger pools of potential study recruits, and thus play a larger role in the growth of chain-referral samples. To our knowledge, the notion that participants’ venue affiliations shape their abilities to recruit other participants has not been tested directly.

The question that we examine here is whether MSM’s connectedness to sex venues affects their prospective referral chains (that is, their recruitment of people who recruit other people) net of MSM’s social network size, which is assumed to be the main factor that drives recruitment [1]. There are reasons to believe that venue affiliation and network connectedness are linked. Consistent with the existing practice of using MSM’s personal network sizes as RDS weights are the ideas that MSM (1) are introduced to sex venues by friends and other contacts and (2) form relationships with new contacts in sex markets[32]. On the other hand, the extent of MSM’s sex venue involvement and the sizes of their personal social networks may be unrelated in some cases. For one, the principle of social network “homophily”–or the tendency for individuals to form ties with others who are similar to them—suggests that one can have numerous network ties and yet exhibit little diversity in their sex venue exposure [33, 34]. Conversely, research shows that people who serve as bridges between otherwise poorly connected groups do not always have large networks [35, 36]. These observations suggest that MSM network size and sex venue structure need not be highly correlated. This raises the possibility that the ability of MSM to expand a sample through recruitment of new members may be as closely tied to those MSM’s involvement in sex venues—where they are exposed to larger pools of people whom they might not even know—as it is to those MSM’s personal social networks.

## Methods

### Study design and objectives

This analysis draws on data from the first wave of the uConnect study (n = 622), completed in 2014. uConnect was implemented by a team of epidemiologists, public health, and social scientists who have expertise in HIV research and prevention, social networks, and sexual behavior in minority communities. It focuses on risk behaviors and health in the lives of Younger Black MSM (YBMSM) in South Chicago. South Chicago and adjacent neighborhoods constitute the largest Black community area in the United States, and include the highest HIV incidence rates in Chicago [37]. Participants completed a questionnaire about their demographic and background information, social network characteristics, community involvement, sex and sex-drug risk/reduction practices, and STI/HIV testing/treatment. The Survey Research Lab at the University of Chicago conducted pilot interviews and cognitive testing of questions. Study participants completed written informed consent. The Institutional Review Boards at the University of Chicago and NORC at the University of Chicago approved the research protocols.

### Sample recruitment and interviewing

RDS implementation began by selecting a diverse profile of YBMSM to serve as seeds. The research team began by gathering a group of about twenty community partners to help identify potential seeds in the community. Seeds were recruited using a variety of techniques, including posts on web sites, Facebook postings, and at college campuses, health clinics, and community events. In all, 38 productive seeds, comprising 6.7% of the final sample, were recruited. This excludes 24 initial interviewees who did not recruit any participants. (None of the results reported here depend on whether these non-productive seeds are included.) The median number of referrals was 3. Among all respondents, the distribution of the number of successful referrals was 0 (6.4%), 1 (44.3%), 2 (24.4%), 3 (15.1%), 4 (5.9%), 5 (2.5%) and 6 (1.4%) yielding maximum referral chains of 13. All of the 38 seeds and subsequent recruits were interviewed. At the end of these interviews, each participant was trained on recruiting other YBMSM using six coupons with unique ID numbers. Each participant was offered $60 for their participation in the interview and $20 for each of their recruits. We study the final referral network, which contains a total of 598 YBMSM, including 38 productive seeds and 560 subsequent (“prospective”) recruits.

Interviews with each of the participants were conducted in private offices on the University of Chicago campus by trained interviewers. Interviews were conducted using Computer Aided Personal Interviewing (CAPI). Some sections of the interview were self-administered. The interview itself involved a variety of questions and activities, yielding an average interview length of 138 minutes.

### Study variables

The dependent variables measure the extent to which a given participant’s recruitment efforts impacted the eventual uConnect sample. This is measured in terms of the nature of referral chains that stemmed from a participant’s recruits. We are not merely interested in whether the respondent was able to recruit participants. Rather, we are interested in whether the referral chains that were generated by participants expanded in the manner preferred by RDS. We measure this in two ways: 1) the total number of RDS recruits in a given respondent’s downstream (i.e., prospective) recruitment chain—that is, how many participants were recruited by the respondent, his recruits, and their subsequent recruits (observed range: 0 to 137); and 2) the length of the longest recruitment chain that stems from the respondent (observed range: 0 to 13).

We examine how social network size and the range of sexual/social venues in which MSM are involved relates to these two measures. Network size is measures as the total number of Black MSM that a given respondent reported knowing (range: 0 to 200). To measure sexual/social venue range, we draw on respondents’ reports of how often, during the past 12 months, they had gone to four different types of venues to “meet or socialize with other men.” The four types included clubs/bars, gyms, adult bookstores/bathhouses and House/Ball spaces. For each type of venue, we asked where these venues were located. Options (“all that apply”) included south side, north side, west side, east side, south side suburbs, or other. We multiplied the number of the four types of venues by the number of the six geographic identifiers to calculate overall sexual/social venue range.

We control for several other factors that may impact referral chain growth, including: 1) at what point (i.e., recruitment “wave”) the participant was recruited in the referral chain, operationalized using a continuous measure indicating wave and a squared term to allow for non-linearity; 2) the participant’s age; 3) the participant’s ethnicity; 4) whether he was a resident of the south side or a suburb in the south side; 5) indicators of his sexual orientation (gay, bisexual, or other non-gay orientation); and 6) a count of the number of geographic regions in Chicago (range 0 to 6, as above) in which he had attempted to meet men in public places (e.g., public parks) during the past 12 months. To reduce endogeneity associated with our main predictors, we also include two other measures of social connectedness to this population: 7) the number of sex partners with whom the participant reported having sex during the last six months (range: 0 to 100); and 8) an indicator of whether he had used social media or hook-up application (e.g., Facebook) during the past 24 hours).

### Statistical analyses

Because the dependent variables are counts, we model them using negative binomial regression. Negative binomial models predict count-related outcomes and include a parameter, σ^2^, that accounts for overdispersion (high variation) in the dependent variable [38]. These analyses are restricted to participants who had valid data on all variables, resulting in a final sample of 567 YBMSM. The main analyses use RDS person-level weights based on the personal social network size of each respondent as described in Gile[39]. To assess sensitivity to RDS weighting, however, supplemental analyses are conducted using Volz-Heckathorn RDS-II weights (S1 Table) and also using no weights at all (S2 Table) [5]. All analyses are conducted using Stata 12.1 (StataCorp LP, College Station, Texas).

## Results

**Fig 1:**
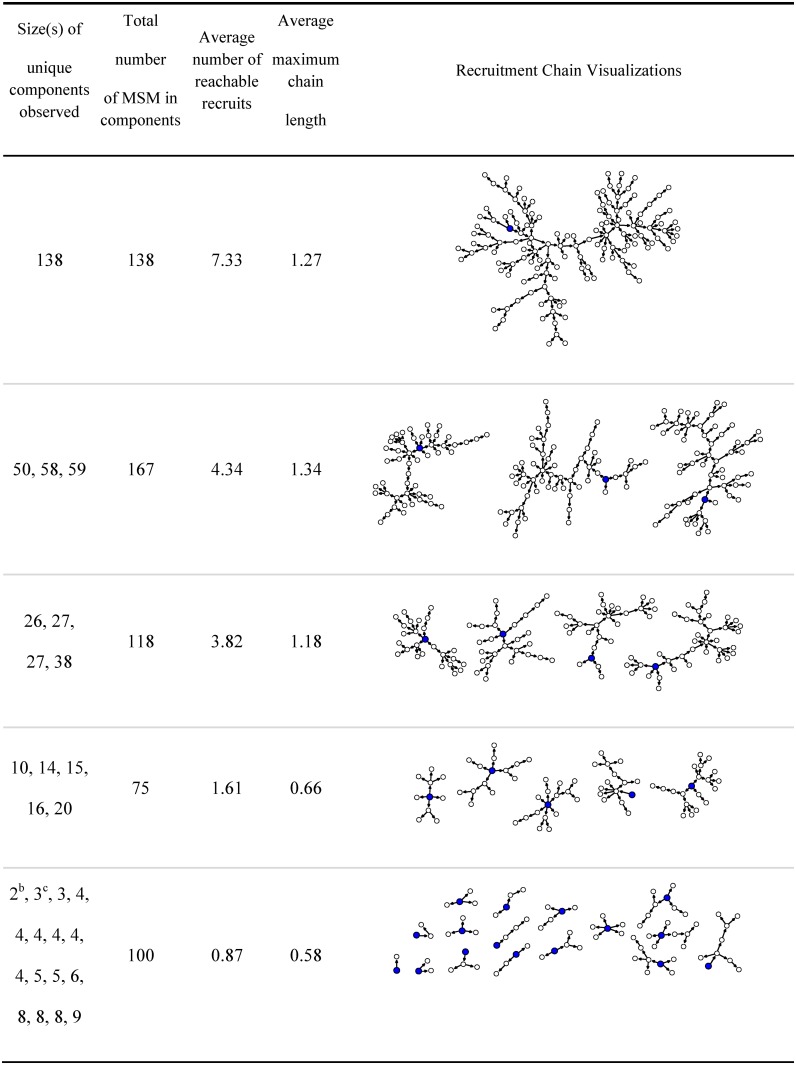
Recruitment networks from uConnect RDS sample of 598 Chicago MSM, 2013-2014^a^.

Characteristics of the uConnect sample are reported in Table 1, and uConnect’s RDS referral chains are represented in Fig 1. On average, seeds generated 12.8 additional participants in the uConnect study through their direct recruitment efforts and/or via the efforts of the subsequent recruits in their referral chains. Non-seed recruits generated, on average, 3.5 subsequent recruits. (This number is low partly due to the fact that 57.4% of non-seed participants did not recruit anyone, including those who did not have time to distribute their coupons within the study’s recruitment time window.) A total of 186 participants generated more than one recruit in their referral chain. The length of the longest referral chain averaged 2.8 among seeds and .97 among other participants. Of the 45.7% of the sample who recruited at least one participant, the average chain length was 2.7, as 54.1% of these people yielded chains containing at least two waves of participants. These recruitment efforts resulted in several long referral chains—with 473 participants (83.4% of the sample) being included in referral networks that contained at least 10 people.

**Table 1:** Descriptive characteristics of MSM in the uConnect study, 2013–2014^a^.

	Seeds (N = 38)		Recruits (N = 529)	
Measure	Mean or No.	*(SD or %)*	Mean or No.	*(SD or %)*
*Dependent variables*				
Overall referral network size	12.84 (25.64)		3.46 (12.53)	
Referral chain length	2.78 (3.37)		.97 (2.02)	
*Independent variables*				
Recruitment wave^b^	1.00 (0.00)		5.64 (3.27)	
Age^b^	23.42 (2.73)		22.60 (3.11)	
Ethicity				
Non-Hispanic *(referrent)*	36	96.00	506	94.62
Hispanic	2	4.00	23	5.38
Residence				
Southside *(referrent)*	36	89.57	449	83.68
Other (e.g.," south side suburbs")	2	10.43	80	16.32
Sexual orientation				
Gay *(referrent)*	27	68.71	354	64.51
Bisexual	10	29.89	140	28.35
Other non-gay	1	1.40	35	7.14
Number of sex partners, last 6 months	4.18 (6.42)		3.55 (6.29)	
Number of black MSM known^b^	6.80 (35.49)		4.21 (17.99)	
Uses social media				
Yes *(referrent)*	30	72.46	347	58.63
No	8	27.54	182	41.37
Meets MSM in outdoor/public spaces?				
No *(referrent)*	19	62.30	305	61.45
Yes	19	37.70	227	38.55
Social/Sexual venue range^b^	1.89 (2.09)		1.30 (1.60)	

Abbreviations: MSM, men who have sex with men; SD, standard deviation; RDS, respondent-driven sampling.

^a^ Estimates do not include respondents who did not receive any coupons, and who are thus not in the main analysis. All mean and percentage estimates are adjusted using Gile’s (2011) person-level RDS sequential sampling weights.

^b^ Values are means (standard deviations).

On average, seeds reported an average network size of 6.8, and other participants reported an average of 4/2. Seeds reported involvement in 1.9 types of sex venues, on average, and other recruits reported involvement in an average of 1.3 types of venues. Overall, 409 (72.1%) reported involvement in at least one type of sex venue, and 222 (39.2%) reported involvement in more than one type of venue. The most popular venues involved clubs or bars on the north side of Chicago (52.2%), the second most popular venues were House/Ball venues on the South Side (24.0%), and the third involved south side clubs/bars (19.4%).

**Fig 2:**
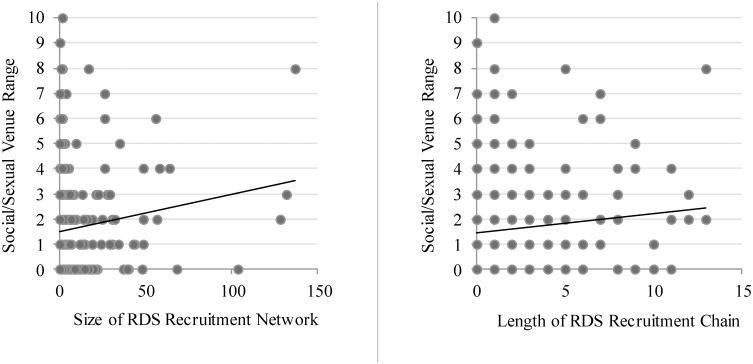
Bivariate associations between the range of MSM’s social/sexual venue affiliation and both the total size of their prospective recruitment network (left panel) and the length of the longest chain in their recruitment network (right panel).

Bivariate regression analyses (not shown) provide mixed marginal evidence of an association between personal network size and recruitment. Personal networks size is not significantly associated with the rate of recruit referrals (IRR = 1.020, *p* = .130), and it is marginally positively associated with the length of recruitment chains (IRR = 1.017, *p* = .053). The number of venue types in which respondents were involved is more significantly associated with impact on RDS referral chain growth. In a bivariate model (not shown), each additional venue type is associated with a 24.1% increment in the rate of network recruit referral (*p* = .006). This association is depicted in the scatterplot shown in the left panel of Fig 2. Likewise, each additional venue type is associated with a 14.5% increment in the length of recruitment chains (*p* = .015). This is depicted in the scatterplot in the left panel of Fig 2.

**Fig 3:**
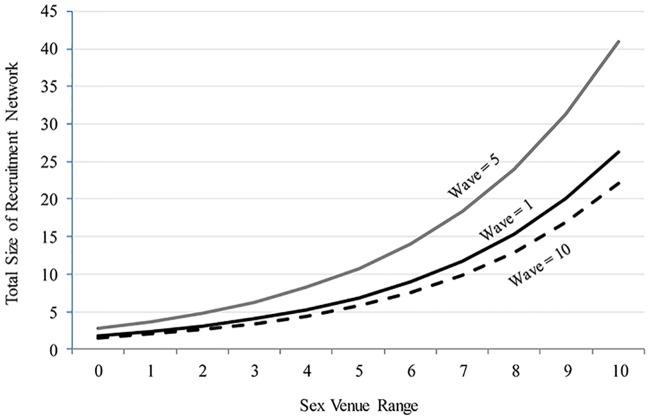
Predicted number of prospective recruits in uConnect participants’ RDS referral networks, by sex market exposure and recruitment wave, net of other factors, based on estimates reported in model 1 of Table 2.

Neither of the associations between personal network size and measures of recruitment success are significant in the multivariate analysis. The associations between venue range and recruitment success, however, remain statistically significant. Table 2 reveals that each additional venue type is associated with a 30.7% increment in the number of recruits (*p* < .001). The multivariate association is illustrated in Fig 3. For example, a YBMSM who was interviewed in the fifth wave and who reported no sex venue involvement is projected to have about three downstream recruits, while someone in the same wave who reported involvement in five types of venues is projected to have about eleven downstream recruits. YBMSM who were enrolled in the middle of the study (e.g., wave 5) had a greater impact on the growth of the referral network than either those recruited early on (wave = 1) or late in the study (wave = 10).

**Table 2:** Incidence rate ratios from multivariate negative binomial regression models predicting (1) total size and (2) maximum chain length of the prospective RDS recruitment networks of MSM in the uConnect study, 2013–2014 (N = 567)^a^.

	Model 1: Total Size of Prospective RDS Recruitment Network	Model 2: Length of Longest Prospective RDS Recruitment Chain
Predictor	IRR (95% CI)	IRR (95% CI)
Recruitment wave	1.31 (1.01, 1.70)	1.00 (.83, 1.22)
Recruitment wave (squared)	0.97 (.95, .99)	1.00 (.98, 1.01)
Age	0.97 (.90, 1.05)	1.01 (.95, 1.06)
Hispanic ethnicity	2.13 (.85, 5.32)	1.42 (.68, 2.97)
Non-southside residence	0.72 (.37, 1.40)	0.77 (.46, 1.31)
Bisexual orientation (vs. gay)	1.28 (.72, 2.29)	1.28 (.84, 1.95)
Other non-gay orientation (vs. gay)	0.40 (.21, .77)	0.78 (.46, 1.30)
Number of sex partners, last 6 months	1.03 (.97, 1.10)	1.02 (.97, 1.08)
Number of black MSM known	1.02 (1.00, 1.05)	1.01 (1.00, 1.03)
Uses social media	1.72 (1.04, 2.83)	1.22 (.85, 1.77)
Meets MSM in outdoor/public spaces	0.53 (.33, .84)	0.75 (.53, 1.05)
Social/Sexual venue range	1.31 (1.13, 1.51)	1.15 (1.03, 1.27)
Intercept	1.97 (.27, 14.44)	0.69 (.17, 2.82)

Abbreviations: RDS, respondent-driven sampling; IRR, incidence rate ratio; CI, confidence interval; MSM, men who have sex with men.

^a^ Applied only to respondents who were provided at least one coupon to distribute. All models are adjusted using Gile’s (2011) person-level RDS sequential sampling weights. Models that employ the Volz-Heckathorn (2008) RDS-II weights yield similar results (S1 Table).

**Fig 4:**
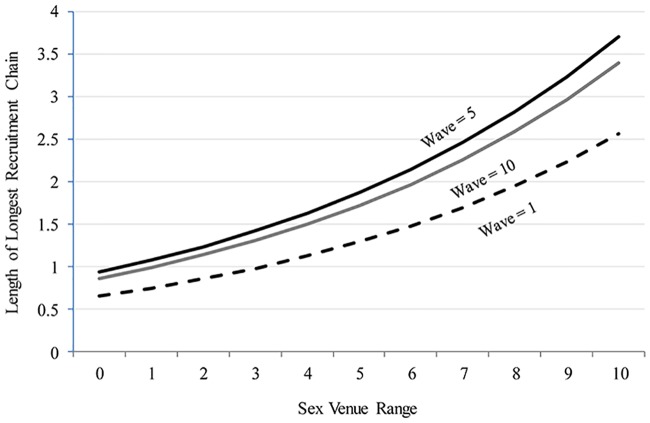
Predicted maximum chain length in uConnect participants’ RDS referral networks, by sex market exposure and recruitment wave, net of other factors, based on estimates reported in model 2 of Table 2.

Table 2 also shows that each additional venue type is associated with a 14.7% increment in the total number of recruits (*p* = .010). This is illustrated in Fig 4. A YBMSM who was interviewed in the fifth wave and who reported no venue involvement, for example, is projected to have a prospective chain of length of about one, while a participant who reported involvement in five venue types is projected to have a chain of length of about two. These results hold when using an alternative(5) RDS person-level weight (S1 Table) as well as (marginally) when no weight is used (S2 Table).

## Discussion

There is still much to learn about the link between study participants’ social connectedness and the success of chain-referral recruitment efforts in hidden populations. The findings of the present study suggest not only that personal network size is not always a useful estimate of participants’ impact on prospective recruitment, but also that exposure to non-specific (perhaps unknown) others through a wide-ranging variety of community venues may be a more useful predictor. Young black MSM who were affiliated with a wider variety of sexual/social venues in different communities not only yielded a larger number of prospective recruits, they also generated longer referral chains. These findings hold net of numerous other significant predictors of YBMSM’s impact on RDS referral chains, including at which wave participants were added to the study, the participant’s age, sexual orientation, their use of social media, their tendency to seek MSM in public places, and, most importantly, the size of their social networks.

These findings have important implications for studies of at-risk populations. Because longer referral chains yield larger and more diverse samples, these results suggest that participants who have more diverse venue affiliations may also generate samples that produce less biased estimates [1, 12]. Chain-referral studies of hidden populations should remain mindful of not only how the sizes of participants’ personal social networks (i.e., the number of others in the target population whom they know) shape their impact on recruitment, but also how participants’ exposure to *broader pools of potential contacts* shape recruitment. Our analyses provide some evidence that this more generalized form of connectedness (involvement in a range of venues) may even have a larger impact on sample growth than does personal social network size. Post-estimation tests reveal that this difference in magnitude is statistically significant with respect to both the size of a participant’s overall referral network (χ^2^ = 11.1, *p* > .001) and the length of his longest referral chain (χ^2^ = 5.4, *p* < .03). Finally, we should note that the finding that participants’ use of online social media to find sexual partners is significantly associated with the total number of prospective recruits (IRR = 1.716, *p* < .05). This finding is also consistent with the notion that social connectedness behaviors that link participants to more generalized pools of potential (weak) MSM contacts are more consequential for subsequent recruitment than is personal network size.

This study has several limitations. For one, the data were self-reported. Response bias is possible in the form of over- or under-inflation of participants’ social network sizes. Such bias, however, would likely be similar across participants and it would be difficult to determine whether it would behave differently from response biases that are related to participants’ venue exposure. Estimating one’s network size may be a more cognitively challenging process than responding to fixed items regarding affiliation with types of sexual/social venues. Relatedly, our measure of venue range may underrepresent the true range of potential types of venues with which YBMSM are involved, and may also suffer from underreporting due to social desirability bias. And we should underscore that while both the measure of network size and venue range may capture exposure to a variety of MSM in the community, they do not capture other key parts of the recruitment process, such as the ability of participants to recruit people who are likely to redeem the coupons. Finally, these findings are drawn from a sample that was observed within a specific geographic area. The impact of venues on other populations’ recruitment efforts may depend on local variability in the presence of venues. Regardless, YBMSM represent a population at greatest risk of HIV in the United States and maximizing recruitment efficiency in this group can provide valid estimates and engage larger numbers of YBMSM in research and downstream HIV prevention interventions.

These findings should inform selection criteria for seeds in future chain-referral RDS studies, and may impact the parameters that are utilized for the weighting of RDS, which currently focus on personal network size. The focus on sexual/social venue involvement, as an affiliation—as opposed to an individual-based network approach [18, 40, 41]–suggests the need for alternative or supplemental weighting schemas. We recommend that careful analyses be conducted to determine whether estimates that are derived using more conventional estimators such as the Volz-Heckathorn [5] estimator and the Gile [39] estimator are sensitive to participants’ venue affiliations. The development of more efficient referral chains has the potential to decrease the costs of RDS studies, reduce bias in estimates, and engage more at-risk community members around HIV prevention research and intervention.

## Supporting information

S1 Table Incidence rate ratios from multivariate negative binomial regression model predicting (1) total size and (2) maximum chain length of the prospective RDS recruitment networks of MSM in the uConnect study, 2013–2014 (N = 567)^a^. (DOCX)

S2 Table Incidence rate ratios from unweighted multivariate negative binomial regression model predicting (1) total size and (2) maximum chain length of the prospective RDS recruitment networks of MSM in the uConnect study 2013–2014 (N = 567)^a^. (DOCX)

## References

[1] Heckathorn DD. Respondent-driven sampling: a new approach to the study of hidden populations. Soc Probl. 1997;44:174–199.

[2] Penrod J, Preston DB, Cain RE, Starks MT. A discussion of chain referral as a method of sampling hard-to-reach populations. Journal of Transcultural Nursing. 2003;14(2):100–107. doi: 10.1177/1043659602250614 12772618 10.1177/1043659602250614

[3] Heckathorn DD. Respondent-driven sampling II: deriving valid population estimates from chain-referral samples of hidden populations. Soc Probl. 2002;49(1):11–34.

[4] Salganik MJ, Heckathorn DD. Sampling and estimation in hidden populations using respondent-driven sampling. Socio Methodol. 2004;34(1):193–240.

[5] Volz E, Heckathorn DD. Probability based estimation theory for respondent driven sampling. Journal of Official Statistics. 2008;24(1):79–97.

[6] Wang J, Carlson RG, Falck RA, Siegel HA, Rahman A, Li L. Respondent-driven sampling to recruit MDMA users: a methodological assessment. Drug Alcohol Depen. 2005;78(2):147–157. 10.1016/j.drugalcdep.2004.10.011 15845318

[7] Magnani R, Sabin K, Saidel T, Heckathorn D. Review of sampling hard-to-reach and hidden populations for HIV surveillance. AIDS. 2005;19(Suppl 2):S67–72. 10.1097/01.aids.0000172879.20628.e1 15930843

[8] Malekinejad M, Johnston LG, Kendall C, Kerr LRFS, Rifkin MR, Rutherford GW. Using respondent-driven sampling methodology for HIV biological and behavioral surveillance in international settings: a systematic review. AIDS Behav. 2008;12(4 Suppl):S105–30. doi: 10.1007/s10461-008-9421-1 18561018 10.1007/s10461-008-9421-1

[9] McCreesh N, Frost S, Seeley J, Katongole J, Tarsh MN, Ndunguse R, et al Evaluation of respondent-driven sampling. Epidemiology. 2012;23(1):138–47. doi: 10.1097/EDE.0b013e31823ac17c 22157309 10.1097/EDE.0b013e31823ac17c PMC3277908

[10] Morineau G, Bollen LJ, Syafitri RI, Nurjannaj N, Mustikawati DE, Magnani R. HIV prevalence and risk behaviours among injecting drug users in six indonesian cities implications for future HIV prevention programs. Harm Reduct. J. 2012;9:37–43. doi: 10.1186/1477-7517-9-37 22943438 10.1186/1477-7517-9-37 PMC3494521

[11] Rudolph AE, Gaines TL, Lozada R, Vera A, Brouwer KC. Evaluating outcome-correlated recruitment and geographic recruitment bias in a respondent-driven sample of people who inject drugs in tijuana, Mexico. AIDS Behav. 2014;18(12):2325–37. doi: 10.1007/s10461-014-0838-4 24969586 10.1007/s10461-014-0838-4 PMC4401495

[12] Gile KJ, Handcock MS. Respondent-driven sampling: An assessment of current methodology. Sociol Methodol. 2010;40(1):285–327. doi: 10.1111/j.1467-9531.2010.01223.x 22969167 10.1111/j.1467-9531.2010.01223.x PMC3437336

[13] Goel S, Salganik MJ. Assessing respondent-driven sampling. P Natl Acad Sci USA. 2010;107(15):6743–6747. 10.1073/pnas.1000261107 PMC2872407 20351258

[14] Johnston LG, Malekinejad M, Kendall C, Iuppa IM, Rutherford GW. Implementation challenges to using respondent-driven sampling methodology for HIV biological and behavioral surveillance: field experiences in international settings. AIDS Behav. 2008;12(1):131–141. 10.1007/s10461-008-9413-1 18535901

[15] Lu X, Bengtsson L, Britton T, Camitz M, Kim BJ, Thorson A, et al The sensitivity of respondent-driven sampling. J Roy Stat Soc A Sta. 2012;175(1):191–216.

[16] Kuhns LM, Kwon S, Ryan DT, Garofalo R, Phillips G, Mustanski BS. Evaluation of respondent-driven sampling in a study of urban young men who have sex with men. J Urban Health. 2015;92(1):151–167. doi: 10.1007/s11524-014-9897-0 25128301 10.1007/s11524-014-9897-0 PMC4338125

[17] Reisner SL, Mimiaga MJ, Johnson CV, Bland S, Case P, Safren SA, et al What makes a respondent-driven sampling “seed” productive? Example of finding at-risk Massachusetts men who have sex with men. J Urban Health. 2010;87(3):467–479. doi: 10.1007/s11524-010-9439-3 20354911 10.1007/s11524-010-9439-3 PMC2871093

[18] Frost SDW. Using sexual affiliation networks to describe the sexual structure of a population. Sex Transm Infect. 2007;83(suppl 1):i37–i42. 17664363 10.1136/sti.2006.023580

[19] Fujimoto K, Williams ML, Ross MW. Venue-based affiliation networks and HIV risk-taking behavior among male sex workers. Sex Transm Dis. 2013;40(6):453–8. doi: 10.1097/OLQ.0b013e31829186e5 23677019 10.1097/OLQ.0b013e31829186e5 PMC3675278

[20] Garcia J, Muñoz-Laboy M, Parker R, Wilson PA. Sex markets and sexual opportunity structures of behaviorally bisexual Latino men in the urban metropolis of New York City. Archives of Sex Behav. 2014;43:597–606. 10.1007/s10508-013-0072-6 PMC4565493 23479357

[21] Laumann EO, Ellingson S, Mahay J, Paik A, Youm Y. eds. The Sexual Organization of the City. Chicago: University of Chicago Press; 2005.

[22] Mauk D, Perry A, Muñoz-Laboy M. Exploring the desires and sexual culture of men who have sex with male-to-female transgender women. Archives of Sex Behav. 2013;42:793–803. 10.1007/s10508-013-0079-z 23572267

[23] Muñoz-Laboy M, Parker R, Perry A, Garcia J. Alternative frameworks for examining Latino male bisexuality in the urban space: a theoretical commentary based on ethnographic research in Rio de Janeiro and New York. Sexualities. 2013;16:501–522.

[24] Binson D, Woods WJ, Pollack L, Paul J, Stall R, Catania JA. Differential HIV risk in bathhouses and public cruising areas. American J Public Health. 2001;91(9):1482–1486. 10.2105/ajph.91.9.1482 PMC1446808 11527785

[25] Grov C. HIV risk and substance use in men who have sex with men surveyed in bathhouses, bars/clubs, and on Craigslist.org: venue of recruitment matters. AIDS Behav. 2012;16(4):807–817. doi: 10.1007/s10461-011-9999-6 21748276 10.1007/s10461-011-9999-6 PMC5826651

[26] Horvath KJ, Bowen AM, Williams ML. Virtual and physical venues as contexts for HIV risk among rural men who have sex with men. Health Psychol. 2006;25(2):237–242. doi: 10.1037/0278-6133.25.2.237 16569116 10.1037/0278-6133.25.2.237

[27] Niekamp A-M, Mercken LAG, Hoebe CJPA, Dukers-Muijrers NHTM. A sexual affiliation network of swingers, heterosexuals practicing risk behaviours that potentiate the spread of sexually transmitted infections: a two-mode approach. Soc Networks. 2013;35(2):223–236.

[28] Oster AM, Wejnert C, Mena LA, Elmore K, Fisher H, Heffelfinger JD. Network analysis among HIV-infected young black men who have sex with men demonstrates high connectedness around few venues. Sex Transm Dis. 2013;40(3):206–212. doi: 10.1097/OLQ.0b013e3182840373 23403601 10.1097/OLQ.0b013e3182840373 PMC4945956

[29] Thiede H, Jenkins RA, Carey JW, Hutheson R, Thomas KK, Stall RD, et al Determinants of recent HIV infection among Seattle-area men who have sex with men. Am J Public Health. 2009;99(S1):S157–S164. 18445808 10.2105/AJPH.2006.098582 PMC2724937

[30] Parsons JT., Halkitis PN. Sexual and drug-using practices of HIV-positive men who frequent public and commercial sex environments. AIDS Care. 2002;14(6):815–826. doi: 10.1080/0954012021000031886 12511214 10.1080/0954012021000031886

[31] Youm Y. A sociological interpretation of emerging properties in STI transmission dynamics: walk-betweenness of sexual networks. Sex Transm Infect. 2010;86(Suppl 3):iii24–iii28. 21098054 10.1136/sti.2010.044008

[32] Cornwell B, Behler R. Sexual behavior and social networks In Wright JD, ed. International Encyclopedia of the Social and Behavioral Sciences. 2nd ed Amsterdam: Elsevier;2015.

[33] Laumann EO, Gagnon JH, Michael RT, Michaels S. The Social Organization of Sexuality: Sexual practices in the United States. Chicago: University of Chicago Press; 1994.

[34] McPherson M, Smith-Lovin L, Cook JM. Birds of a feather: Homophily in social networks. Annu Rev of Socio. 2001;27:415–444.

[35] Burt RS. Structural Holes: The Social Structure of Competition. Cambridge: Harvard University Press; 1992.

[36] Roberts SGB, Dunbar RIM, Pollet TV, Kuppens T. Exploring variation in active network size: Constraints and ego characteristics. Soc Networks. 2009;31(2):138–146

[37] Chicago Department of Public Health. HIV/STI Surveillance Report, 2014. Chicago, IL: City of Chicago; 2014.

[38] McCullagh P, Nelder JA. Generalized Linear Models. 2nd ed New York: Chapman and Hall;1989.

[39] Gile KJ. Improved inference for respondent-driven sampling data with application to HIV prevalence estimation. J Am Statistical Association. 2011;106(493):135–146 (2011).

[40] Borgatti SP, Everett MG. Network analysis of 2-mode data. Soc Networks. 1997;19(3)243–269.

[41] Latapy M, Magnien C, Vecchio ND. Basic notions for the analysis of large two-mode networks. Soc Networks. 2008;30(1):31–48.

