# Supplementary material for: Social venue range and referral chain impact: Implications for the sampling of hidden communities
Source: PLoS One. 2017 Aug 23;12(8):e0181494. doi: 10.1371/journal.pone.0181494 (PMC5568219; doi:10.1371/journal.pone.0181494)
Supplement: S2 Table — (DOCX) [file pone.0181494.s002.docx]

**Appendix Table A2. Incidence Rate Ratios from Unweighted Multivariate Negative Binomial Regression Model Predicting (1) Total Size and (2) Maximum Chain Length of the Prospective RDS Recruitment Networks of MSM in the uConnect Study 2013-2014 (N = 567)^a^**

|  | **Model 1: Total Size**  **of Prospective RDS**  **Recruitment Network** |  | **Model 2: Length of**  **Longest Prospective RDS**  **Recruitment Chain** |
| --- | --- | --- | --- |
| Predictor | IRR (95% CI) |  | IRR (95% CI) |
| Recruitment wave | 1.15 (.90, 1.47) |  | 0.93 (.78, 1.11) |
| Recruitment wave (squared) | 0.98 (.96, 1.00) |  | 1.00 (.99, 1.01) |
| Age | 1.02 (.95, 1.09) |  | 1.03 (.98, 1.08) |
| Hispanic ethnicity | 1.31 (.48, 3.53) |  | 1.15 (.58, 2.29) |
| Non-southside residence | 0.56 (.31, 1.02) |  | 0.67 (.44, 1.03) |
| Bisexual orientation (vs. gay) | 0.87 (.55, 1.39) |  | 1.02 (.72, 1.39) |
| Other non-gay orientation (vs. gay) | 0.49 (.21, 1.17) |  | 0.93 (.51, 1.70) |
| Number of sex partners, last 6 months | 1.00 (.96, 1.03) |  | 1.00 (.97, 1.03) |
| Number of black MSM known | 1.00 (.99, 1.02) |  | 1.00 (.99, 1.01) |
| Uses social media | 1.52 (.97, 2.37) |  | 1.25 (.91, 1.71) |
| Meets MSM in outdoor/public spaces | 0.58 (.39, .88) |  | 0.80 (.60, 1.08) |
| Social/Sexual venue range | 1.19 (1.06, 1.34) |  | 1.07 (.99, 1.17) |
| Intercept | 2.34 (.42, 13.08) |  | 0.77 (.23, 2.58) |

Abbreviations: RDS, respondent-driven sampling; IRR, incidence rate ratio; CI, confidence interval; MSM, men who have sex with men.

^a^ Applied only to respondents who were provided at least one coupon to distribute.
